# Specialist psychiatric health care utilization among older people with intellectual disability – predictors and comparisons with the general population: a national register study

**DOI:** 10.1186/s12888-020-02491-6

**Published:** 2020-02-17

**Authors:** G. Ahlström, A. Axmon, M. Sandberg, J. Hultqvist

**Affiliations:** 1 Department of Health Sciences, Faculty of Medicine, Lund University, Lund, Sweden; 2 EPI@LUND (Epidemiology, Population studies, and Infrastructures at Lund University), Lund University, Lund, Sweden

**Keywords:** Aging, Learning disabilities, Co-morbidities, Diagnoses, Mental disorders, Mental retardation, Psychiatric health care, Register data

## Abstract

**Background:**

People with intellectual disability (ID) face considerable barriers to accessing psychiatric health care, thus there is a risk for health disparity. The aims of the present study were 1) to compare specialist psychiatric health care utilization among older people with ID to that with their age peers in the general population, taking into account demographic factors and co-morbidities associated with specialist psychiatric health care utilization and 2) to determine a model for prediction of specialist psychiatric health care utilization among older people with ID.

**Material and methods:**

We identified a national cohort of people with ID (ID cohort), aged 55+ years and alive at the end of 2012 (*n* = 7936), and a referent cohort from the general population (gPop cohort) one-to-one matched by year of birth and sex. Data on utilization of inpatient and outpatient specialist psychiatric health care, as well as on co-morbidities identified in either psychiatric or somatic specialist health care, were collected from the National Patient Register for the time period 2002–2012.

**Results:**

After adjusting for sex, age, specialist psychiatric health care utilization the previous year, and co-morbidities, people in the ID cohort still had an increased risk of visits to unplanned inpatient (relative risk [RR] 1.95), unplanned outpatient (RR 1.59), planned inpatient (RR 2.02), and planned outpatient (RR 1.93) specialist psychiatric health care compared with the general population. Within the ID cohort, increasing age was a predictor for less health care, whereas psychiatric health care the previous year predicted increased risk of health care utilization the current year. As expected, mental and behavioral disorders predicted increased risk for psychiatric health care. Furthermore, episodic and paroxysmal disorders increased the risk of planned psychiatric health care.

**Conclusions:**

Older people with ID have a high need for psychiatric specialist health care due to a complex pattern of diagnoses. Further research needs to investigate the conditions that can explain the lesser psychiatric care in higher age groups. There is also a need of research on health care utilization among people with ID in the primary health care context. This knowledge is critical for policymakers’ plans of resources to meet the needs of these people.

## Background

It is increasingly recognized that people with intellectual disability (ID) face considerable barriers to accessing psychiatric health care [1–5]. Thus there is a risk for health disparity [6], if there are population-specific differences in access to health care or in health outcomes [7]. The reported prevalence of psychiatric disorders among people with ID varies greatly. In two systematic reviews the reported prevalence ranged from 3.9 to 46.3% [8] and from 13.9 and 75.2% [9], respectively**.** Most of the variation was due to differences in diagnostic criteria, and in how the specific samples were obtained. Albeit of these divergent figures, psychiatric disorders are more prevalent among adults with ID than in the adult general population [8, 9]. An increased life expectancy among people with ID [10, 11], together with a positive association between age and psychiatric disorders in this group [1, 12–14], results in an increasing number of older people with ID and concurrent psychiatric disorders. Research regarding psychiatric health care utilization among older people with ID is scarce [15], however, and the present study addresses this knowledge gap.

A longitudinal study carried out in Ontario, Canada, reported that one-third of the total inpatient stays (somatic and psychiatric) among people with ID was made up by inpatient psychiatric care. However, the study lacked comparisons with a general population cohort [15]. A further British cross-sectional study, found that those with ID who received specialist psychiatric health care were more likely to be older (> 30 years) [16]. Again, no comparison was made with the general population. Nevertheless, the results of another study in Canada [17], using national data, found that people with ID were more likely to have two or more hospitalizations during the investigated year compared to the general population. All these studies included adult people with ID, but did not focus on older people specifically. Thus, investigating utilization of psychiatric health care among older people is of relevance.

Older people with ID and psychiatric disorders are a particularly complex and vulnerable group, having higher rates of somatic problems than individuals with ID without psychiatric disorders [18, 19]. Using Swedish national register data, we have previously shown that older people with ID had higher psychiatric health care utilization (in- and outpatient specialist health care) compared to the general population [20]. However, in these comparisons, we did not take differences in co-morbidities into account. In Sweden, people with ID have access to the same psychiatric health care as the general population, and the rights to an equal health care are based on needs stipulated in the Swedish Health and Medical Services Act [21]. Comparing utilization of psychiatric health care among older people with ID to that in the general population, while taking into account factors associated with psychiatric health care utilization, is therefore of importance.

There is a knowledge gap regarding factors that may predict psychiatric health care utilization among older people with ID. Identifying such predictors is of importance for service development and for preventive actions. Previous research regarding people with ID suggests that age [17, 22], sex [23] and psychiatric diagnosis [22, 24, 25] are factors of importance, with the addition of somatic diagnoses [19]. However, to the best of our knowledge, no previous studies have investigated psychiatric and somatic co-morbidities simultaneously.

To conclude, increased knowledge of differences in psychiatric health care utilization between older people with ID and older people in the general population, as well as identifying predicting factors for psychiatric health care utilization in older people with ID, can help health care staff understand these issues and provide guidance for strategies for services and mental health improvement among older people with ID. The aims of the present study were therefore:

1) To compare specialist psychiatric health care utilization among older people with ID to that of their age-peers in the general population, taking into account demographic factors and co-morbidities.

2) To determine a model for prediction of specialist psychiatric health care utilization among older people with ID.

## Methods

### Study cohorts

In Sweden, people with disabilities are entitled to services and support from the municipality to manage their daily lives. The support available is regulated in the Swedish Act Concerning Support and Service for Persons with Certain Functional Impairments (Swedish abbreviation: LSS), [26]. The law applies to three separate groups of people with significant and long-term functional disabilities, whereof people with ID and/or autism spectrum disorder (ASD) comprise group 1. All the provided support is recorded in the LSS register at the Swedish National Board of Health and Welfare.

We identified all people in group 1 with at least one measure of support according to the LSS during 2012, aged 55+ years and alive at the end of that year. These comprised the ID cohort (*n* = 7936), i.e. we used receiving support according to the LSS as a proxy for having ID. A referent cohort from the general population, one-to-one matched to the ID cohort by sex and year of birth (gPop cohort, *n* = 7936) was supplied by Statistics Sweden, the government agency responsible for official statistics.

Each cohort comprised 3609 (45%) women and 4327 (55%) men. Their mean age on December 31, 2012, was 64 years (range 55–96).

### Health care utilization

The Swedish National Patient Register (NPR) contains data on visits to inpatient and outpatient specialist health care. For each visit, one primary and up to 21 secondary diagnoses are recorded, along with information on whether the visit was planned (i.e. the appointment was made beforehand) or unplanned, and from which clinic the patient was discharged.

Data were collected on visits to clinics within the psychiatric health care sector from the NPR for the time period 2002–2012. For each person and year, data were aggregated into four dichotomous variables, indicating whether the person had at least one of the following type of psychiatric health care during that year: planned inpatient, planned outpatient, unplanned inpatient, and unplanned outpatient.

### Co-morbidities

In order to be able to adjust for co-morbidities in cohort comparisons (aim 1) and include co-morbidities as potential predictors for psychiatric health care utilization (aim 2), we collected information on all diagnoses recorded 2002–2012 from the NPR. In doing this, we considered both primary and secondary diagnoses, and made no restrictions to clinic (i.e. also diagnoses in somatic health care were included). Diagnoses of ID (F7 in ICD-10), ASD (F84), and Down syndrome (Q90) were excluded.

### Statistical analysis

Potential impact of co-morbidities, previous psychiatric health care utilization (i.e. psychiatric health care utilization the year before), sex, age, and cohort affiliation (aim 1 only) on the four types of psychiatric health care utilization (planned/unplanned inpatient/outpatient) was evaluated using generalized linear models (GLMs) with Poisson distribution and log link, thus estimating relative risks (RRs) with 90% confidence intervals (CIs). Data were aggregated by year, and calendar year was used to indicate repeated measures. A similar method was employed to evaluate aim 2, with the exceptions that only data from the ID cohort were used which means that cohort affiliation was not included in the models.

Co-morbidities were investigated on block level, as defined in ICD-10. Yearly inpatient psychiatric health care utilization was categorized as 0, 1, or 2+ visits, whereas yearly outpatient psychiatric health care utilization was categorized as 0, 1, 2–5, 6+ visits. Age was aggregated to 10-year intervals up to 80 years old, and the remaining persons to the category 80+ years.

Both aims and all four types of psychiatric health care utilization were investigated using similar procedures:

1. **Bivariate** models were used to investigate possible associations between different diagnoses (i.e. co-morbidities) and specialist psychiatric health care utilization. This was done for all diagnostic blocks for which at least five people had a diagnosis. All diagnoses with *p* < 0.1 were carried forward to multivariate models in the next step.
2. **Unadjusted multivariate** models were used to assess the possible associations between different diagnoses and specialist psychiatric health care utilization, taking into account all diagnoses found to associate with health care utilization in bivariate models (i.e. step 1). Those diagnoses with *p* < 0.1 in these multivariate models were carried forward to the adjusted multivariate models in the next step.
3. **Adjusted multivariate** models were used to assess possible associations between different diagnoses and specialist psychiatric health care utilization, taking into account all diagnoses found to associate with health care utilization in multivariate models (i.e. step 2) as well as cohort affiliation (aim 1 only), sex, age, and psychiatric health care utilization the previous year.

The predictive ability of the models from the analyses within the ID cohort was assessed using AUC (area under the curve) and displayed using ROC (receiver operating characteristics) curves.

All analyses were made in IBM SPSS statistics Version 25.

## Results

### Cohort comparisons

In the adjusted multivariate models, i.e. after adjusting for sex, age, previous psychiatric health care utilization the year before, as well as all relevant (i.e. with *p* < 0.1 in the unadjusted multivariate models) co-morbidities, people in the ID cohort still had an increased risk of unplanned inpatient (RR 1.95, 90% CI 1.70–2.24), unplanned outpatient (RR 1.59, 1.37–1.84), planned inpatient (RR 2.02, 1.58–2.58), and planned outpatient (RR 1.93, 1.78–2.11) psychiatric health care compared to the gPop cohort.

### Predictive models within the ID cohort

**Fig. 1:**
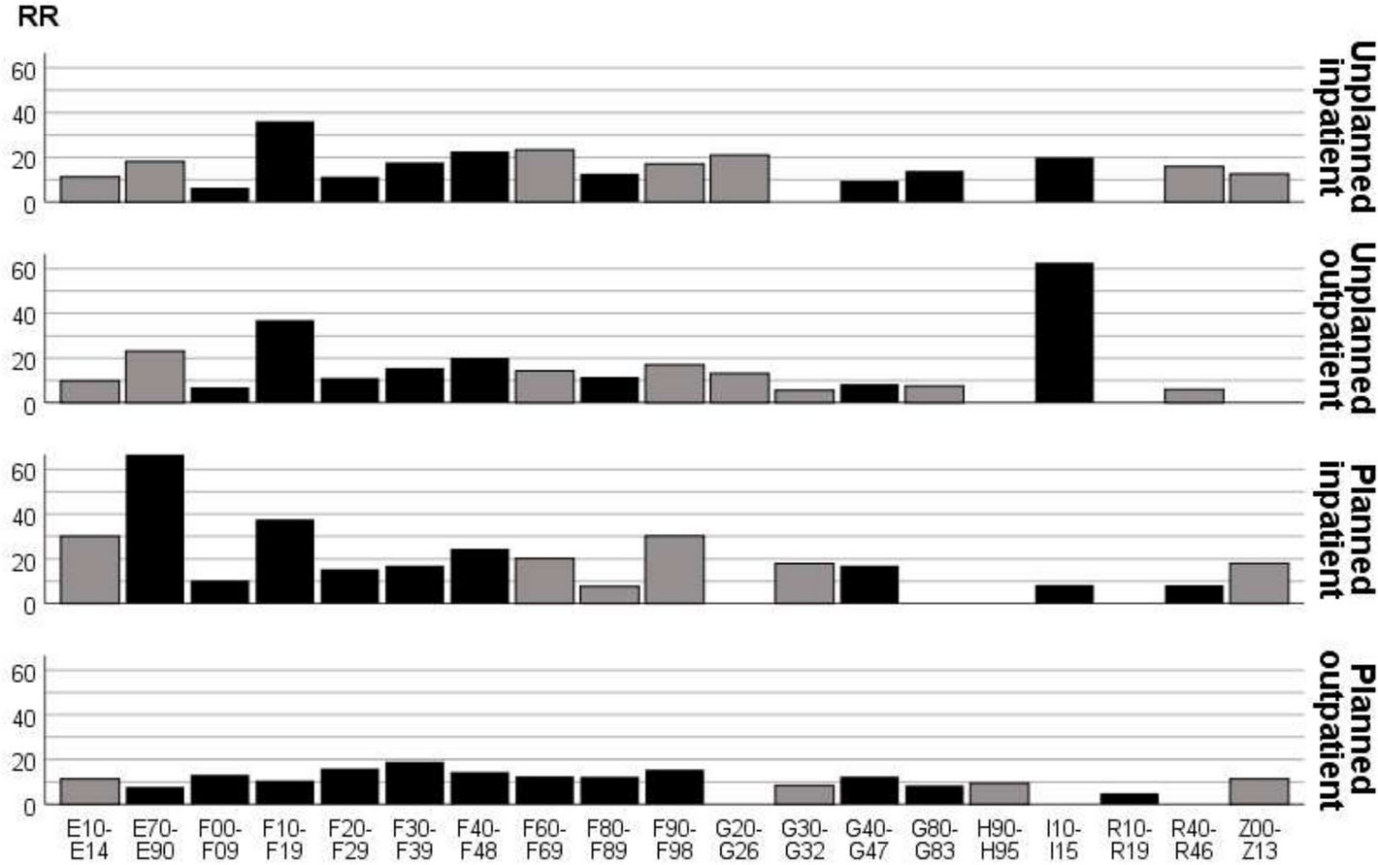
Statistically significant (p < 0.10) relative risks (RR) for diagnosis (ICD-10 codes) vs psychiatric health care utilization among 7936 older people with intellectual disability. Black bars indicate diagnoses still statistically significant (*p* < 0.10) in multivariate models including all diagnoses associated with each respective type of psychiatric health care. *ICD-10 codes: E10-E14 Diabetes mellitus; E70-E90 Metabolic disorders; F00-F09 Organic, including symptomatic, mental disorders; F10-F19 Mental and behavioural disorders due to psychoactive substance use; F20-F29 Schizophrenia, schizotypal and delusional disorders; F30-F39 Mood [affective] disorders; F40-F48 Neurotic, stress-related and somatoform disorders; F60-F69 Disorders of adult personality and behaviour; F80-F89 Disorders of psychological development (excluding F84 Pervasive developmental disorders); F90-F98 Behavioural and emotional disorders with onset usually occurring in childhood and adolescence; G20-G26 Extrapyramidal and movement disorders; G30-G32 Other degenerative diseases of the nervous system; G40-G47 Episodic and paroxysmal disorders; G80-G83 Cerebral palsy and other paralytic syndromes; H90-H95 Other disorders of ear; I10-I15 Hypertensive diseases; R10-R19 Symptoms and signs involving the digestive system and abdomen; R40-R46 Symptoms and signs involving cognition, perception, emotional state and behaviour; Z00-Z13 Persons encountering health services for examination and investigation*

Co-morbidities that were associated with health care utilization in the bivariate and unadjusted multivariate models are presented in Fig. 1.

In the adjusted multivariate models, i.e. when including all diagnoses with *p* < 0.10 in the unadjusted multivariate model together with age, sex and psychiatric health care utilization the previous year, increasing age was consistently a predictor for less health care, whereas no differences were found between men and women (Table 1). Moreover, psychiatric health care utilization the previous year carried an increased risk of health care utilization the current year. As expected, diagnoses of mental and behavioral disorders (Chapter V in ICD-10, excluding F7 and F84) carried increased risk of psychiatric health care. However, also hypertensive diseases (I10-I15) were associated with increased risk of unplanned inpatient and outpatient psychiatric health care, and episodic and paroxysmal disorders (G40-G47) with increased risk of planned inpatient and outpatient psychiatric health care.

**Table 1:** Final models, ID cohort (RR with 90% CI, bold marks statistically significance on the 10% level, NI = not included in the model)

		Unplanned health care		Planned health care	
		Inpatient	Outpatient	Inpatient	Outpatient
Demographics					
50–59 vs < 50 years		0.84 (0.70–1.02)	0.88 (0.68–1.14)	**0.66** (**0.46**–**0.95**)	1.04 (0.92–1.16)
60–69 vs < 50 years		**0.49** (**0.38**–**0.62**)	**0.54** (**0.40**–**0.73**)	**0.43** (**0.28**–**0.66**)	**0.83** (**0.73**–**0.94**)
70–79 vs < 50 years		**0.24** (**0.16**–**0.36**)	**0.23** (**0.14**–**0.37**)	**0.26** (**0.13**–**0.52**)	**0.53** (**0.43**–**0.65**)
80+ vs < 50 years		**0.12** (**0.04**–**0.38**)	**0.09** (**0.02**–**0.45**)	**0.17** (**0.04**–**0.78**)	**0.31** (**0.18**–**0.54**)
Men vs women		0.91 (0.79–1.06)	1.04 (0.88–1.23)	0.88 (0.68–1.14)	0.97 (0.90–1.03)
Diagnoses					
E70-E90: Metabolic disorders		NI	NI	**2.37** (**1.06**–**5.29**)	0.65 (0.37–1.15)
F00-F09: Organic, including symptomatic, mental disorders		1.28 (0.90–1.81)	**1.59** (**1.04**–**2.42**)	**1.79** (**1.05**–**3.06**)	**1.51** (**1.33**–**1.71**)
F10-F19: Mental and behavioral disorders due to psychoactive substance use		**2.31** (**1.66**–**3.21**)	**3.12** (**2.16**–**4.51**)	1.56 (0.98–2.50)	NI
F20-F29: Schizophrenia, schizotypal and delusional disorders		**1.73** (**1.35**–**2.21**)	**2.00** (**1.46**–**2.75**)	**2.11** (**1.38**–**3.23**)	**1.83** (**1.63**–**2.05**)
F30-F39: Mood [affective] disorders		**2.15** (**1.69**–**2.74**)	**2.30** (**1.76**–**3.01**)	**1.56** (**1.03**–**2.36**)	**1.82** (**1.64**–**2.01**)
F40-F48: Neurotic, stress-related and somatoform disorders		**2.03** (**1.57**–**2.61**)	**2.11** (**1.52**–**2.93**)	**1.88** (**1.25**–**2.83**)	**1.35** (**1.22**–**1.50**)
F60-F69: Disorders of adult personality and behavior		NI	NI	NI	**1.19** (**1.01**–**1.41**)
F80-F89: Disorders of psychological development		**1.78** (**1.33**–**2.39**)	**1.79** (**1.21**–**2.67**)	NI	**1.24** (**1.06**–**1.46**)
F90-F98: Behavioral and emotional disorders with onset usually occurring in childhood and adolescence		NI	NI	NI	**1.19** (**1.03**–**1.38**)
G40-G47: Episodic and paroxysmal disorders		1.25 (0.95–1.65)	1.28 (0.80–2.05)	**1.87** (**1.17**–**2.99**)	**1.26** (**1.08**–**1.46**)
G80-G83: Cerebral palsy and other paralytic syndromes		1.85 (0.74–4.66)	NI	NI	0.82 (0.60–1.13)
I10-I15: Hypertensive diseases		**2.39** (**1.30**–**4.38**)	**2.87** (**1.13**–**7.30**)	2.07 (0.80–5.36)	NI
R10-R19: Symptoms and signs involving the digestive system and abdomen		NI	NI	NI	0.95 (0.66–1.38)
R40-R46: Symptoms and signs involving cognition, perception, emotional state and behavior		NI	NI	0.96 (0.54–1.72)	NI
Health care utilization previous year					
Unplanned inpatient	1 vs 0	**9.43** (**7.06**–**12.59**)	**2.14** (**1.55**–**2.96**)	**6.98** (**4.45**–**10.95**)	**1.27** (**1.13**–**1.43**)
	2+ vs 0	**11.45** (**8.14**–**16.11**)	**1.59** (**1.05**–**2.39**)	**6.54** (**3.86**–**11.09**)	1.04 (0.88–1.22)
Unplanned outpatient	1 vs 0	**1.30** (**1.00**–**1.68**)	**4.65** (**3.43**–**6.31**)	**2.21** (**1.39**–**3.51**)	**1.33** (**1.16**–**1.52**)
	2–5 vs 0	1.05 (0.78–1.40)	**4.19** (**2.87**–**6.11**)	**1.83** (**1.16**–**2.90**)	**1.24** (**1.05**–**1.46**)
	6+ vs 0	1.00 (0.53–1.91)	**5.55** (**2.60**–**11.84**)	**3.07** (**1.36**–**6.91**)	0.98 (0.65–1.46)
Planned inpatient	1 vs 0	**2.17** (**1.68**–**2.79**)	**1.92** (**1.34**–**2.75**)	**8.03** (**4.91**–**13.13**)	**1.42** (**1.20**–**1.69**)
	2+ vs 0	**1.74** (**1.17**–**2.60**)	**1.82** (**1.18**–**2.82**)	**7.37** (**4.25**–**12.76**)	1.21 (0.91–1.62)
Planned outpatient	1 vs 0	**2.06** (**1.63**–**2.61**)	**3.09** (**2.30**–**4.15**)	**1.76** (**1.23**–**2.53**)	**14.06** (**12.48**–**15.84**)
	2–5 vs 0	**1.72** (**1.31**–**2.27**)	**2.22** (**1.57**–**3.14**)	**1.74** (**1.14**–**2.66**)	**15.27** (**13.33**–**17.49**)
	6+ vs 0	**1.75** (**1.15**–**2.64**)	**2.20** (**1.30**–**3.72**)	1.50 (0.90–2.50)	**14.85** (**12.14**–**18.15**)

**Fig. 2:**
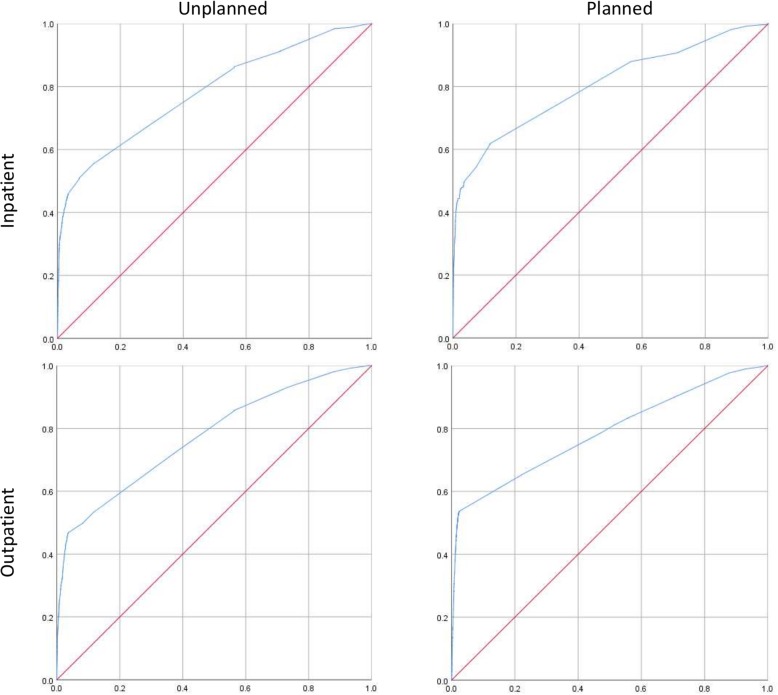
ROC-curves displaying the predictive ability of the models evaluated for the ID cohort (x-axis displays 1-specificty and y-axis displays sensitivity)

All four adjusted multivariate models predicted psychiatric health care utilization better than chance (Fig. 2).

## Discussion

The results of the present study showed that even after adjusting for sex, age, previous psychiatric health care utilization, as well as all relevant co-morbidities, people in the ID cohort still had a higher psychiatric health care utilization than their age-peers in the general population.

To the best of our knowledge, this is the first study to investigate psychiatric health care utilization among older people with ID while also considering demographics and co-morbidities. Thus, our study is likely the first to establish that even when taking all the above mentioned factors into account, older people with ID had higher psychiatric care utilization than their age-peers in the general population. However, being first also limits the possibilities of comparing our results with previous research. Previous studies mainly had a descriptive, and not a predictive, research design. The studies lacked a matched comparison sample from the general population [15, 27], was based on self-report data [28], was based on the data collected from parents of adults with ID [29], had a cross-sectional study design [16], or used regional and not national data [15].

The results from the cohort comparisons cannot necessarily be interpreted straightforward. On one hand, planned health care may be a result of follow-up and proper monitoring of known health issues. In this light, the increased risk of planned inpatient and outpatient care among people with ID may have a positive interpretation, i.e. that psychiatric disorders are well treated among people with ID. On the other hand, unplanned health care is a likely indicator of unmet health care needs. This too was higher among people with ID than in the general population, suggesting the opposite interpretation, i.e. that psychiatric disorders are not well treated among people with ID. Although the comparisons are adjusted for all co-morbidities found in the NPR, we cannot exclude the possibility that the two cohorts differ further in diagnoses made only in primary care. Thus, the differences in psychiatric health care utilization may be explained by disorders unmeasured in this study. Further research need to be performed to entangle the complex picture of health care utilization for psychiatric co-morbidities among people with ID.

Within the ID cohort only, psychiatric health care 1 year predicted the same type of care also the following year. Regarding planned psychiatric health care this could be interpreted as continuity of care and that needs for psychiatric health care were met. However, that similar results were found for unplanned inpatient health care is a cause for concern. It seems reasonable to assume that unplanned inpatient psychiatric health care occasions would warrant comprehensive discharge planning and follow-up in outpatient psychiatric health care or primary healthcare. Previous research has shown, however, that effective coordination of psychiatric health care across service providers for people with ID who have mental disorders may be inadequate [30]. Furthermore, discharge from inpatient psychiatric health care is delayed for people with ID [31], which indicates a need for improvement of care-paths and strategies for discharge. To sum up, whether our findings about unplanned psychiatric care are indications of unmet psychiatric health care needs, or if older people with ID do not have access to planned psychiatric health care on the same terms as the general population is an urgent question for future research. This knowledge is critical for policymakers’ plans of resources to meet the needs of the target group.

The question of possible unmet health care needs is a concern for psychiatric health care services but also for managers and care staff of community support and services for the target group. As indicated by the LSS register, daily activities at a day center or a disability adapted place of work is the most common form of support for people with ID younger than 64 years old, and for people 65 years and older the most common form of support is housing with special services [32]. Thus, the majority of people with ID are closely monitored by care staff who should help them come into contact with psychiatric health care when needed. We have previously shown, however, that people with ID living in special housing during the entire study period (11 years) were less likely to receive psychiatric health care than those who lived in special housing during a part of the study period, or not at all [20]. Barriers for accessing psychiatric health care among people with ID have been identified on both personal and organizational levels [33]. A tendency among care and health care staff to explain negative behavior or symptoms in terms of ID hampers recognition of mental disorders in the target group, which is referred to as diagnostic overshadowing [34]. Difficulty by staff in ID services to recognize the presentation of psychiatric disorders in people with ID [35], and a lack of knowledge about appropriate level of care and how to navigate in the system, can restrict access to psychiatric health care for the caretaker, i.e. the person with ID [36, 37]. Another important issue is that behavioral changes may indicate underlying somatic illness or conditions [38]. Thus staff in ID services need to be able to differentiate this from mental disorders. Training staff in ID services to raise their awareness, knowledge and competence in these respects is therefore of importance [35], so that they are able to better advocate for people with ID and mental disorders when in contact with psychiatric health care [39, 40]. The need to advocate for people with ID is stressed by a study which found that the patients with ID were more likely to have shorter consultations with the physician and to have lower continuity of care with the same physician in primary health care [41].

The present study focused on specialist psychiatric health care utilization (i.e. secondary health care) and not on primary health care. A large proportion of people in the general population in Sweden who have common psychiatric disorders (e.G. *major* depression, anxiety disorders) are followed up, or treated only, in primary health care [42]. It is unknown whether this also applies to people with ID.

A report from the Swedish National Board of Health and Welfare [43] showed that older people in the general population who had mental disorders, or mental ill health, mainly accessed primary health care for treatment, and to a lesser extent specialist psychiatric health care. If the gPop cohort had a higher primary health care utilization for mental health concerns than the ID cohort, this could be one possible explanation for the higher risk of psychiatric specialist health care in the ID cohort in the present study. Thus, research in a primary health care context concerning health care utilization among older people with ID and psychiatric disorders could complement our findings, and add to the understanding of psychiatric health care utilization in this group. Another possible explanation for the higher risk of psychiatric specialist health care in the ID cohort is that people with ID have more complex and severe mental disorders than the general population, which require specialist psychiatric health care [16, 31]. On the other hand, people with ID are less likely to be referred to specialist psychiatric health care than the general population [44, 45]. It is likely that the policy of referring to specialist psychiatric health care is affected by clinical experiences and vary between physicians, care units and different geographical settings.

In a previous study we have shown that there were sex differences regarding psychiatric diagnosis in older people with ID [23], which could have an impact on psychiatric health care utilization. The results of the present study showed, however, that there was no difference between men and women regarding psychiatric health care utilization when including co-morbidities in the analysis. Thus psychiatric health care utilization seem to be based on need rather than gender. This is positive in light of that we have previously shown gender differences in specialist somatic [46] as well as psychiatric [20] health care among older people with ID.

Even though life expectancy is increasing among people with ID [11], it is still shorter than in the general population [47]. In the general population somatic and psychiatric comorbidity is associated with premature death [48, 49]. Older people with ID have shown to have more co-morbidities, including psychiatric ill-health, than the general population [18, 50]. When investigating the ID cohort solely in the present study, however, psychiatric health care decreased in the oldest group. This finding raises a concern considering the increasing risk for ill-health with increasing age [18, 19]. Since the reason for the decrease is unknown this knowledge gap should be addressed in future research.

People with ID and psychiatric disorders have poorer somatic health than people with ID without psychiatric disorders [18, 19]. The present study investigated possible associations between different diagnoses and psychiatric health care utilization. The results showed, as expected, that diagnoses of mental and behavioral disorders (Chapter V in ICD-10) carried increased risk of psychiatric health care. Moreover, episodic and paroxysmal disorders (G40-G47) were associated with increased risk of planned inpatient and outpatient psychiatric health care. These disorders include neurological diseases such as epilepsy. A study [18] exploring multi-morbidity in older people with ID found that the most prevalent multi-morbidity pattern was mental health in combination with neurological disease. Nonetheless, that study did not explore neurological disease as a predictor for psychiatric health care utilization, and to the authors’ knowledge there are no such studies.

Hypertensive diseases (I10-I15) were associated with increased risk of unplanned inpatient and outpatient psychiatric health care. A diagnosis of hypertension is commonly established in clinical practice through at least two separate elevated blood pressure outcomes, with a few days or a week between the measurements [51, 52]. Also, longtime registration of blood pressure at home under 2 days and nights are common when severe hypertension is suspected. In this study, we do not know how the measurements were made before registration in the NPR register. If the diagnosis is based only on a single measurement at the visit in psychiatric or somatic health care, the diagnosis may be stress-related caused by the test environment or procedure [53]. This weakness of the present study indicates the need for further research on hypertension as a predictor of psychiatric health care utilization.

Several studies have concentrated on psychiatric health care needs of people with ID, and how to address those needs [36, 37, 54, 55]. This is an important area for research especially given that it is linked to psychiatric health care utilization [17, 56, 57]. Moss et al. [37] concluded that the psychiatric needs of people with ID are complex, and that sustainable improvements of psychiatric health care provision and community support and services need to be guided by an evidence-based approach and coherent policies. Research regarding inpatient health care has shown that staff failed to meet needs for care [58], showed poor or negative attitudes towards people with ID and lacked skills and knowledge regarding ID [59]. These issues are important areas of improvement for providers of health care and commissioners [58]. Furthermore, inpatient psychiatric health care among people with ID could likely be avoided to some extent by meeting more needs in outpatient psychiatric health care/primary health care/community-based services [60]. Accordingly, assessing needs and coordinating services are of importance [37, 61]. During the time period of this study, the Swedish legislation [21, 62] was amended. The legislated requirement is on interaction between health care and social services in the form of Coordinated Individual Plan (CIP), a shared care plan. The purpose of a CIP is to get an overview of complex needs which require simultaneous planning and action by the two principal’s; health care and social services, to assure that the patient’s/client’s needs are met. The implementation of CIP for older people without ID receiving municipality care was a slow process with disparities between municipalities [63]. It is reasonable to assume that this was also true for older people with ID. Future empirical studies examining the use of CIP, and evaluating the effects of coordinate interventions for older people with ID and psychiatric disorders are warranted.

There are several strengths with the present study. These include the use of national registers to identify the study cohorts and collect data on outcomes and potential confounders, large study populations, and a long study period. The latter is particularly important as it allowed us to take previous health care utilization into consideration when building a model to predict future use. However, there are also some weaknesses in the present study. As we had access to specialist health care data only, we could not identify co-morbidities that had been diagnosed in primary care. However, it is reasonable to assume that such co-morbidities are not associated with specialist psychiatric health care and that they would not have been included in the adjusted multivariate models even if they were tried in the bivariate models. Another weakness is the use of LSS support as a proxy for having ID. This may have caused two problems: 1) That we have failed to include people with ID who did not receive LSS support, and 2) that we included people with ASD but without ID. Also, we did not have data on the severity of ID for the majority of the cohort. Although we did find at least one F7-diagnosis (F70-F79) for about a third of the cohort in the NPR data, this group was too small to perform any subgroup analyses, e.g. to investigate the importance of severity of ID. Previous research has shown that ASD and level of ID can be of importance for the use of psychiatric health care [64]. The inability to differentiate subgroups in our analyses may result in that the health care utilization was over- or underestimated for a specific subgroup. Finally, in interpreting the results regarding co-morbidity, it is important to remember that it is a proxy of disease/disorders and not the cause of the health care visit.

## Conclusions

Older persons with ID are more likely to receive all types of psychiatric health care than persons in the general population, even when taking psychiatric disorders and somatic co-morbidities into account. The results indicate the importance of a planned care plan with follow-up for the people with psychiatric health care utilization, especially for those cared for in the previous year. The background to why the oldest have less health care warrants further research. Whether our findings are indications of unmet health care needs is an urgent question for future research. This knowledge is critical for policymakers’ plans of resources to meet the needs of the target group.

## Data Availability

The data in the present study contains sensitive information on a very vulnerable group, i.e. people with ID. Even though the data are anonymized, it contains enough details to enable identification of single individuals. Therefore, in order to approve the study, the Regional Ethical Review Board in Lund made considerable restrictions regarding access to the data. This means we will not be able to provide other researchers with our data. However, as our database is compiled by register data only, other researchers may contact Statistics Sweden and the Swedish National Board of Health and Welfare to get access to the different registers included, and thereby recreate the database.

## Abbreviations

ASD: Autism Spectrum Disorder
AUC: Area under the curve
CI: Confidence interval
GLM: Generalized linear model
GPop cohort: General population cohort
ICD-10: International statistical classification of diseases and related health problems, 10th revision
ID: Intellectual disability
LSS: The act concerning support and service for people with certain functional impairments
NPR: National Patient Register
ROC: Receiver operating characteristics
RR: Relative risk
